# Android avatar improves educational effects by embodied anthropomorphization

**DOI:** 10.3389/frobt.2024.1469626

**Published:** 2025-01-06

**Authors:** Naoki Kodani, Takahisa Uchida, Nahoko Kameo, Kurima Sakai, Tomo Funayama, Takashi Minato, Akane Kikuchi, Hiroshi Ishiguro

**Affiliations:** ^1^ Department of Engineering Science, Osaka University, Osaka, Japan; ^2^ Sociology Department, New York University, New York, NY, United States; ^3^ Hiroshi Ishiguro Laboratories, Advanced Telecommunication Research Institute International, Kyoto, Japan; ^4^ RIKEN, Kyoto, Japan; ^5^ KiQ Inc., Tokyo, Japan

**Keywords:** distance learning, android, avatar, lecture, education

## Abstract

After the COVID-19 pandemic, the adoption of distance learning has been accelerated in educational institutions in multiple countries. In addition to using a videoconferencing system with camera images, avatars can also be used for remote classes. In particular, an android avatar with a sense of presence has the potential to provide higher quality education than a video-recorded lecture. To investigate the specific educational effects of android avatars, we used a Geminoid. an android with the appearance of a specific individual, and conducted both laboratory experiment and large-scale field experiment. The first compared the android avatar lecture with a videoconferencing system. We found that the use of an android avatar for the lecture led to the significantly higher subjective feelings of being seen, feeling more motivated, and focused on the lecture compared to the video lecture. We further conducted a large-scale field experiment with an android avatar to clarify what contributes to such educational effects. The results suggest that the students’ perception of android’s anthroppomorphism and competence has a positive impact, and discomfort has a negative impact on the subjective experence of educational effect. These results indicate the role of embodied anthropomorphization in positive educational experience. The important point of this study is that both the laboratory experiment and the large-scale experiment were conducted to clarify the educational effects of androids. These results support several related studies and are clarified in detail. Based on these results, the potential for the future usage of androids in education is discussed.

## 1 Introduction

In recent years, remote lectures and conferences have increasingly become common in universities and other educational institutions, as well as in companies. In educational institutions, the COVID-19 pandemic certainly served as a trigger to incorporate distance learning as part of the educational experience. Previous research has developed an algorithm that determines how many students can go to school in person with minimal risk for educations such as COVID-19 (Tutsoy, 2021). These studies indicate that distance learning is essential for high-quality education in disaster situations.

The accessibility of distance learning is by no means only useful in disaster situations. Remote learning of various formats has been increasingly implemented in educational institutions in recent decades, enabling students to continue their education even when in-person learning is not feasible for a diverse set of reasons, such as cost, location, or work schedule. In this article we use the term “distance learning” as in Moore et al. (2011); all types of learning methods that do not involve in-person interaction between peers and teachers (Moore et al., 2011). Pregowska et al. summarized the evolution of distance learning, beginning with radio programs and educational content shown using television, cassettes, videotapes, floppy disks, CD-ROMs, the internet, virtual reality (VR), augmented reality, and mixed reality (Pregowska et al., 2021).

Despite its increasing popularity, the literature shows mixed results on the efficacy of distance learning. Pregowska et al. (2021) concluded that although distance learning is a worthwhile alternative, it cannot replace the direct contact between teachers and students for practical knowledge (Pregowska et al., 2021), and Bray et al. found that student evaluations in online distance university of distance learning depended on their ease of computer interaction, social interaction in learning, etc. (Bray et al., 2008).

As Pregowska et al. (2021) have noted, one of the main challenges in distance learning is to establish a connection between an instructor and a student. For this, new technologies such as robots, VR, and the use of online avatars have the potential to enhance the quality of education. as distance learning is increasingly being adopted in educational settings for various reasons, it is even more important to research various distance learning systems and their implementation formats from the perspective of their educational effects. For example, in the Pantelidis’s review article, the advantages of VR include motivation and learning contexts that are difficult to experience in the real world. Its disadvantages, such as high cost, can be overcome as this technology takes root outside the educational field (Pantelidis, 2009).

This article focuses on robots, as they present a unique potential for their use in the educational field (Hoshikawa et al., 2015). The use of robots is known to enhance the communication between teachers and students (Jiro et al., 2021) and the motivation of students (Kashihara, 2019). When using robots for distance learning, it is likely that different types of robots have different educational effects. In this study, we have focused on androids. The studies on androids done to date have focused on issues such as whether the operator identifies with the teleoperated androids (Ilona et al., 2010) and the created androids that mimic individuals, such as the Einstein android (Oh et al., 2006). To the best of our knowledge, no study has been done on investigating the potential of an android for education in detail, and in a more systematic manner.

This study aims to verify the effects of android-based distance learning. Sakamoto et al. developed “Geminoids,” which are android avatars that have the same appearance as specific individuals (Sakamoto et al., 2007) and are usually teleoperated by the person who the android is modeled after (operators were not the model of the Geminoid used in Sakamoto’s experiment). They found that participants felt a stronger presence of the operator when talking to the Geminoid teleoperated by the operator, compared to talking with the operator remotely through a video monitor via a video conference. Inspired by these results, a Geminoid that has the same appearance as that of Prof. Hiroshi Ishiguro of Osaka University was used in our system. A comparison was done of two modes of learning, one in which Prof. Ishiguro gave a lecture through a videoconferencing system and the other, the lecture in which the Geminoid of Prof. Ishiguro gave a lecture.

In Study 1, an experiment was conducted to compare distance learning using an android avatar with that using a video conferencing system to investigate the effectiveness of using androids in distance learning. The android used in this research had the same appearance as Prof. Hiroshi Ishiguro of Osaka University, and so he was speaking in the video for fulfilling the videoconferencing system condition. Based on the results obtained in Study 1, a large-scale field experiment with high school students was conducted in Study 2 using the same Geminoid. Based on the evaluation data of the students for the lecture, a path analysis was conducted to investigate the factors that increase the educational effects of lectures given by the android avatar. Following the experiments in Studies 1 and 2, this study quantitatively evaluated the effects of using android avatars in distance learning, and discuss how android avatars can contribute to higher-quality education in distance learning.

A few previous studies have investigated the use of teleoperated android in education (Hashimoto et al., 2011; Hoshikawa et al., 2015). In education settings, Hoshikawa et al. (2015) have shown that android lectures using the Geminoid could enhance the participants’ perception of the lecturers’ attention to participants. Additionally, the study has shown that android lectures could cause nervousness among the participants, suggesting that the comfort level of the participants is an important factor to investigate when implementing an android in an educational setting. Building on these results, the first contribution of this study is to compare between an android avatar and a videoconferencing system in detail to show the importance of embodiment of a lecture. Additionally, as the second contribution, this study conducted a large-scale field experiment and found that students’ perception of anthropomorphism and competence has a positive impact, and discomfort has negative impact for the subjective educational effects. This study explores the educational effects of an android avatar in both laboratory experiments, revealing the factors that contribute to better an educational experience.

## 2 Related work

Guo et al. researched video-based online classes to investigate engagement of students in these classes and found that it was higher when the instructor’s talking head and slides were displayed in an alternating manner (Guo et al., 2014). From this suggestion, the presence of an instructor may be important for student engagement. Onishi et al. showed that partial embodiment of the remote partner significantly enhances the partner’s social telepresence in the pointing task (Onishi et al., 2016). In particular, they showed that social telepresence was highest when participants could see the face of the remote partner through remote display and their arm movement was embodied by synchronized robotic arms. Thus, the use of robots that partially embody the partner’s bodies may be advantageous for interaction.

Various studies have been also conducted on the use of telepresence robots in workplaces, schools, and educational settings (Tsui et al., 2011; Tanaka et al., 2014; Newhart and Olson, 2017). If the embodiment of a teacher or person by a robot enhances their social presence, the next question is what sort of educational effects the different kinds of robots have. Haring et al. compared androids, humanoid robots, and nonbiomimetic robots and reported that although androids were more likely to be anthropomorphized than other robots before interaction, androids are no longer significantly different from humanoid robots and non-biomimetic robots in post-interaction evaluations (Haring et al., 2016). However, their study did not focus on whether the social presence of a particular persona, i.e., the lecturer themselves, was better enhanced by using androids that significantly resembled that person. In an educational setting, such specific social presence might matter more than the simple telepresence of a (general) remote person, because distance learning, in general, lacks the connection between the particular lecturer and the student as Pregowska et al. (2021) noted.

Velentza et al. conducted a study in which a robot was used as a tutor in a class (Velentza et al., 2021). It showed that the score of learning questionnaires was higher given by a human than given by a robot in the first lesson, but in the second lesson given by a robot, the score was higher than in the first lesson given by a human. Furthermore, they showed that even the first lesson is perceived as more enjoyable given by the robot than by the human. Their experiments were well-designed to show the educational effects of the robots. On the other hand, the robot used in their experiments was a small humanoid robot, and there are insufficient considerations regarding their appearance and other controls such as size comparing them to humans. Thus, it is necessary to conduct experiments with robots that have the same kind of appearance as humans such as androids.

There is a limited amount of research done specially for androids, and most of these studies have focused on the design and production of the appearance and movements of the android, such as its laughter and facial expressions (Ishi et al., 2019; Choi et al., 2011). Hashimoto et al. conducted research on teaching with androids and found that elementary school students were significantly interested in science after a lecture through a remote-controlled android than prior to the lecture, but the effects of using an android were confounded with the effect of simply having a lecture on science (Hashimoto et al., 2011). Although they mentioned performing a comparison with videoconferencing systems as future work, they have yet to conduct such research. Hoshikawa et al. compared the Geminoid lecture and video-conference system in a one-on-one lecture setting. They found that the participants felt that the lecturer attended to the participants more, and made eye-contact to the students more in the android lecture setting. They also found that the Geminoid lecture is more likely to make participants feel nervous, thus pointing out the need for the researchers to attend to the adverse effect of uncanny valley (Mori, 2011) in implementing the android lecture in an educational setting.

Hoshikawa et al. (2015) compared a robot with a video of a person, and showed the importance of human-like functions such as eye contact for the educational experence. The advantage of their study is that the comparative experiments were conducted using Geminoid and the video of the same lecturer the robot is based on. However, they have not conducted detailed investigations of the factors that enhance the educational effects of androids. Another research also studied a class using a Geminoid, but it focused on factors of robot’s evaluations such as seating position and gender differences (Abildgaard and Schärfe, 2012). Their research reveals the influence of external factors other than the Geminoid itself in class. Hence, the relationship between educational effects and android evaluation has not been clarified in detail. Thus, our study further clarifies the effects of using an android avatar in an educational setting. There are various statistical methods for performing detailed studies on data. Guggemos et al. use partial least squares structural modelling to investigate the relationship between the intention to use a robot for learning and the evaluation of the robot such as reliability (Guggemos et al., 2020). Such a model allows for statistical analysis of the effects of paths between items or factors. Therefore, this study investigates the factors that affect the educational effects of an android by using a covariance structure analysis. The quality of the lecture was controlled in this study (i) by conducting the same lecture through video conferencing and through the android for multiple participants (The content of the lecture was same but the timing of slide changing and the timing of speaking was slight differed) and (ii) by using an android avatar of a specific lecturer. In addition, an investigation on whether the embodiment of a persona helps students learn in a non-in-person educational environment was also done. To the best of our knowledge, this is the first study that investigates the effect of embodying a specific lecturer in a controlled experimental setting as well as a field study in a secondary educational setting.

## 3 Android system

**FIGURE 1:**
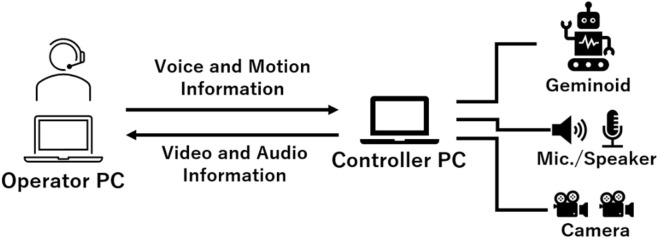
Schematic of the remote control system of the Geminoid.

In this study, an android with a human appearance was used. In particular, a Geminoid that has the same appearance as a specific person was used. The Geminoid was modeled after Prof. Hiroshi Ishiguro of Osaka University and its appearance is shown Figure 1. The system that controlled the android was based on prior research (Minato et al., 2023). The android can give automatic lectures based on prerecorded speech and motions, and can also be operated remotely.

For an automatic lecture, the android was connected to a PC and behaved according to predefined motions. In this case, several android motions were defined in advance and the timing of the expressions was controlled using a controller PC in accordance with the voice, which was emitted from a speaker connected to the PC.

Figure 1 shows the system connection for the remote control. The PC was connected to an android, two cameras, and a microphone speaker that had an echo cancellation function. The PC of the operator received video from the two cameras and audio from the microphone speaker. The operator observed them and operated the android through the interface. The operator’s voice and motion information were sent from their PC to the controller PC.

## 4 Study 1 laboratory experiment on distance lectures

### 4.1 Method

An experiment was conducted to investigate whether lectures given by an android avatar in a lecture setting provide better educational experiences compared to those given through videoconferencing systems used by multiple educational institutions that offer distance learning. Two conditions were compared among the participants: (i) a lecture given by an android that has the appearance of Prof. Hiroshi Ishiguro and (ii) a lecture that used a video of Prof. Hiroshi Ishiguro. After the lecture, the participants were given a surprise test without prior notice of the contents of the lecture and an evaluation of their impressions of the lecture. The surprise test scores and impression evaluation results were used to investigate the differences in the educational effects between the lectures given by the android and using a videoconferencing system. The score of the surprise test (max. nine points) and the impression evaluation (seven-point Lickert scale) are statistically tested for comparing the videoconferencing system condition and the android avatar condition. In other words, the scores are dependent variables, and the condition is independent one. The results are evaluated according to the significant differences between the two conditions.

### 4.2 Condition

**FIGURE 2:**
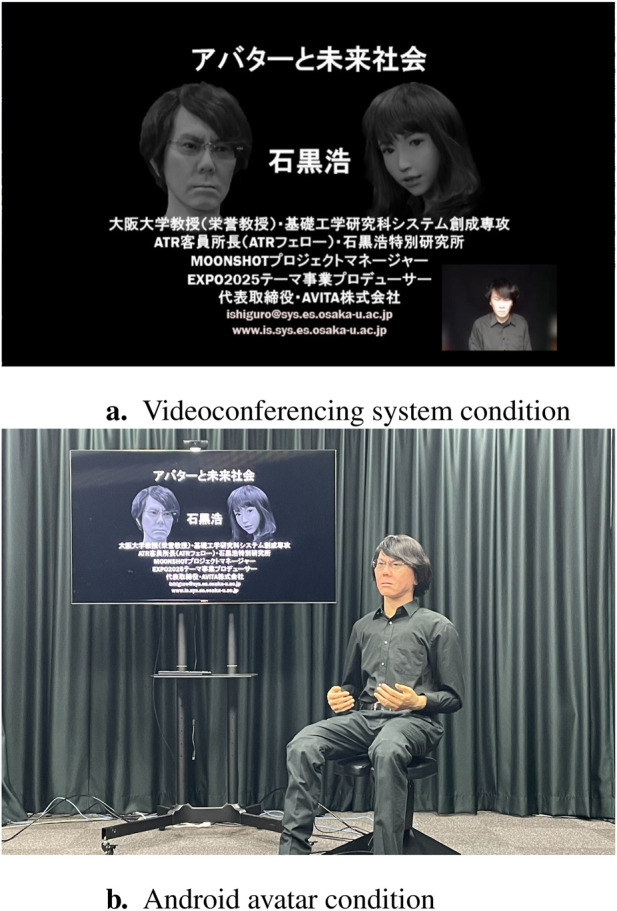
Images of the two experimental conditions. These pictures are images and not of the experiment as it was conducted. **(A)** Videoconferencing system condition. **(B)** Android avatar condition.

**FIGURE 3:**
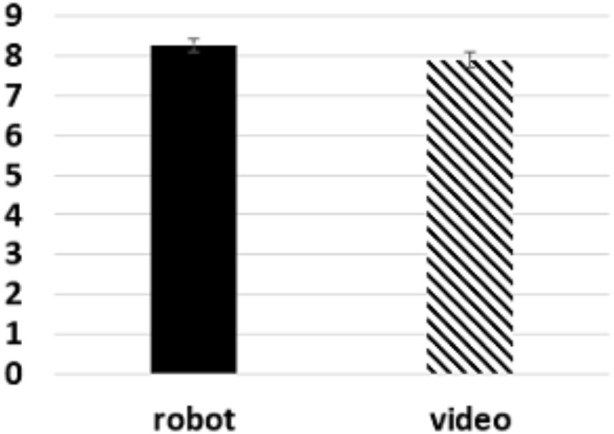
Results of the surprise test.

An experiment was conducted to compare two conditions, namely, a lecture using a video conferencing system and a lecture given by an android avatar in distance learning, in which the speaker participated from a remote location. In the case of the lecture given using a videoconferencing system, a slideshow of the lecture was projected on the screen, and the video of the lecturer speaking was displayed on the lower right corner of the slide show (Figure 3A). In this condition, the video was not a real-time lecture, but a prerecorded video. In the case of the lecture given by android avatar, a slideshow of the lecture was projected on a screen and the android avatar was placed next to it (Figure 2B). The voice was prerecorded, and the motions and eye movements were made in advance based on the prerecorded voice. The title of the lecture was “Avatar and Future Society” and focused on the introduction of avatars and autonomous interactive robots. The lecture lasted approximately 34 min. The participants were told that it was a special lecture by Prof. Hiroshi Ishiguro of Osaka University, and that they were not instructed in advance on whether the lecture would be live or recorded. For both conditions, at the end of the experiment, the participants were informed that the lecture was not live but recorded.

### 4.3 Participants

Sixty two people between the ages of 18 and 60 participated in the experiment (Male = 39, Female = 23, M = 25.40, SD = 9.68): 28 participants in the videoconferencing system condition and 34 participants in the android avatar condition. The participants were randomly assigned to one of the two conditions. To check for any bias between the participants in the two conditions, at the beginning of the experiment, the participants were asked how often they use a computer (FC), how often they watch a science fiction movie (FM), how often they actually see a humanoid robot (FH), and how they are interested in robots (IR) on a 5-point scale. These items are similar to those used in previous study (Kamide et al., 2013). Levene’s test for equality of variances was conducted between in both the conditions and the results showed significant differences in the FC values (FC, p=0.027<0.05 ; FM, p=0.395 ; FH, p=0.948 ; IR, p=0.092 ). Therefore, a t -test with no assumption of equal variances was conducted for FC, which showed no significant difference between the two conditions ( df=45.569 , t=1.066 , p=0.292 ). Further, a t -test conducted by assuming equal variances for FM, FH, and IR showed no significant differences (FM, df=60 . t=0.216 , p=0.830 ; FH, df=60 , t=−0.883 , p=0.065 ; IR, df=60 , t=0.830 , p=0.410 ). Thus, no significant differences were observed among the participants in the two conditions for the items checked in this study.

### 4.4 Measurement

The surprise test was used as a method to comprehensively compare the comprehension, memory, and focus of the participants on the lecture content in the videoconferencing system condition and android avatar condition. The participants were only told that they will answer a brief questionnaire after the lecture but not that a test will be conducted. After the lecture, they were asked to answer nine questions as a test on the contents of the lecture. All questions were multiple-choice questions that examined the participants’ understanding of the lecture, and the participants selected their answers from the four options given for each question. To evaluate the educational effect of the lecture from the participants’ viewpoint, they were also asked to answer four additional questions regarding their subjective assessment of the lecture on a seven-point scale (1:Strongly Disagree, 2:Disagree, 3:Somewhat disagree, 4:Can’t say either way, 5:Somewhat agree, 6:Agree, 7:Strongry agree). The questions were modeled after those used in previous studies on robots and education (Kashihara, 2019; Shimaya et al., 2021; Tomoji et al., 2003). The four questions were as follows: I felt this robot (speaker) was looking at us (LK), I could concentrate on this lecture (FS), I could listen to this lecture without losing motivation until the end (MN), and this lecture was easy to understand (UD).

### 4.5 Result

For the surprise test, each question was scored as one point. The average score was higher for the android avatar condition than in the videoconferencing system condition (Figure 3). Levene’s test for equality of variances was performed, from which an insignificant value (p=0.734) was obtained. Therefore, to deteremine whether there is a difference between the averages of the two conditions, a t -test assuming equal variances was conducted and no significant difference was observed ( t=−1.550 , df=60 , p=0.063 ).

**FIGURE 4:**
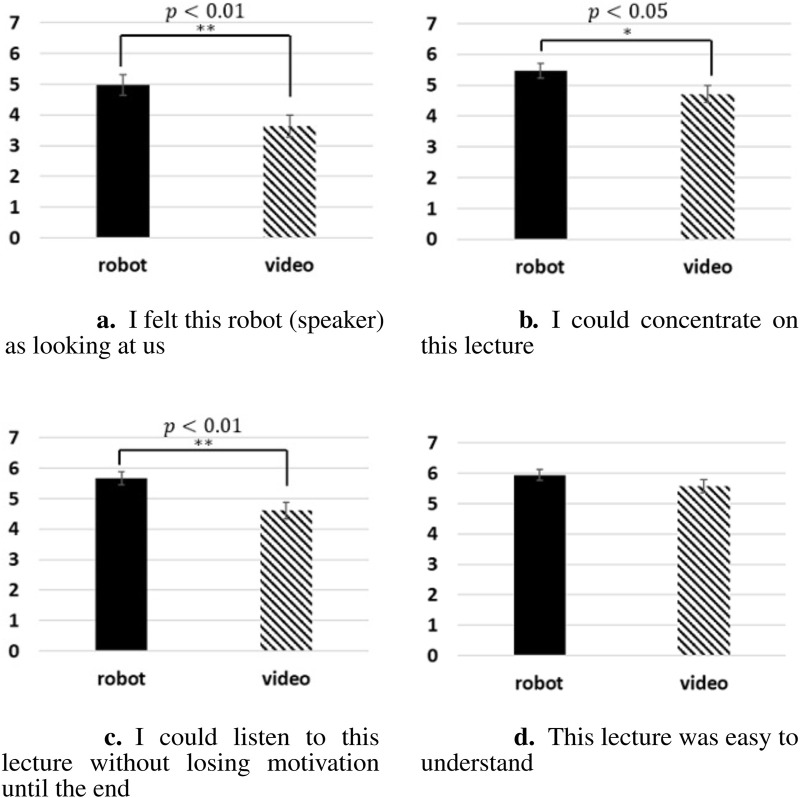
Averaged results of the questionnaire in the lab experiment. **(A)** I felt this robot (speaker) as looking at us. **(B)** I could concentrate on this lecture. **(C)** I could listen to this lecture without losing motivation until the end. **(D)**. This lecture was easy to understand.

The averaged results of the questionnaire are shown in Figure 4 for the videoconferencing system and android avatar conditions. For all four items, the mean value was higher for the android avatar condition than for the videoconferencing system condition. Levene’s test for equality of variances was performed for each item and the difference was not significant (LK, p=0.967 ; FS, p=0.687 ; MN, p=0.393 ; UD, p=0.220 ). Therefore, the t -test was done assuming equal variances for each, from which significant values for LK, FS, and MN and insignificant value for UD were observed (LK, df=60 , t=−2.684 , p=0.009<0.01 ; FS, df=60 , t=−2.112 , p=0.039<0.05 ; MN, df=60 , t=−3.121 , p=0.003<0.01 ; UD, de=60 , t=−1.316 , p=0.193 ). These results suggest that the participants have significantly higher feeling of beeing seen, focused, and motivated in the android avatar condition than in the videoconferencing system condition.

## 5 Study 2 large-scale field experiment

The results of Study 1 confirmed that the lecture given by an android avatar was educationally more effective than the lecture given through a videoconferencing system. Study 2 further explored the factors that contributed to the educational effectiveness based on the participants’ subjective educational experience with the android avatar by conducting a large-scale field experiment in a real secondary educational setting in a high school environment.

### 5.1 Method

#### 5.1.1 Condition

**FIGURE 5:**
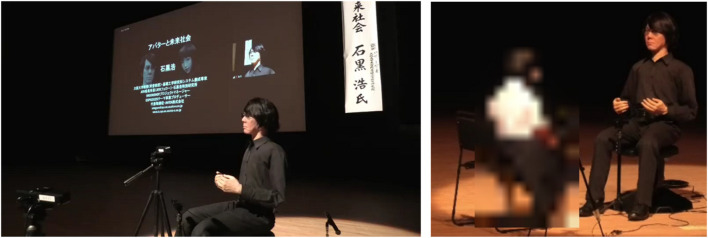
Images of the large-scale field experiment.

A large-scale field experiment was conducted to evaluate the impression of the lecture given by the android avatar to high school students. Similar to Study 1, a Geminoid having the same appearance as Prof. Hiroshi Ishiguro was used as the android, and the lecture was conducted in two parts: a lecture, followed by a question-and-answer (Q&A) session. The lecture was held at the Tottori Prefecture Citizen’s Culture Hall, The Rika Hall (maximum capacity of 2,000 people) in which high school students of one high school in Tottori convened to allow for a large audience. After Prof. Hiroshi Ishiguro was introduced by the principal of the high school, the lecture, titled “Avatar and Future Society” was conducted for approximately 1 hour. Similar to the android avatar condition in Study 1, the lecture in the hall was delivered using the recorded audio, motion, and slideshows. The android was placed on the right-hand side facing the screen. In addition to the presence of the android throughout the lecture and the Q&A session, the android’s face was projected on the screen along with the slideshow to allow for better visibility for the audience in seats further away from the stage; the setup closely resembling any big lecture, e.g., TED talks, where the speaker presents their slides during the lecture and the slides and the upper body of the speaker are displayed simultaneously on large screens (Figure 5 left). During the Q&A session, Prof. Hiroshi Ishiguro himself remotely operated the android in real-time from a remote-control room at Osaka University. To engage in the Q&A session, a chair was set up in front of the android on the stage, and a representative of all high school students sat in a chair and asked questions to the android (Figure 5 right). Questions were also asked by the audience from the audience seats. The operator in this case operated the android’s line of sight and controlled its motions (self-mentioning, bowing, etc.) to compensate for the lack of vision of the entire lecture hall from the remote-control room at Osaka University.

### 5.2 Participants

The lecture was attended by students and parents from Tottori Prefectural Tottori Nishi High School (approximately 900 participants). After the lecture, a voluntary questionnaire survey of the high school students was conducted and valid responses were obtained from 245 students aged 15 to 18 (Male =102 , Female =136 , Unknown =7 , M=16.16 , SD=0.95 ).

### 5.3 Measurement

In this study, our questionnaires for anthropomorphism were prepared referring to several previous studies such as the study in which the impressions of humanoid robots were extensively studied (Kamide et al., 2013). This study also incorporated the measures from the RoSAS (Carpinella et al., 2017), namely, warmth, competence, and discomfort. For investigating the educational effects, the same questionnaire as in Study 1 (Kashihara, 2019; Shimaya et al., 2021; Tomoji et al., 2003) was used. The answers to these questions were recorded on a 5-point scale (1: Strongly Disagree, 2: Disagree, 3: Can’t say either way, 4: Somewhat agree, 5: Strongly agree). The results of all the questionnaires (five point Likert scale) are analyzed to clarify their relationships. The results of the questionnaire measure anthropomorphism, warmth, competence, discomfort, and educational effects. Using SPSS AMOS (a statistical software), a model is created with the raw data of the questionnaire as input. The results are evaluated based on whether the relationship (path on the model) between each latent variable and subscale (answer of the questionnaires) is significant or not.

### 5.4 Result

Table 1 shows the results for each questionnaire item. All items on anthropomorphism exceeded a median of 3. In particular, for the item pertaining to appearance, the average was high, 4.36, with a small standard deviation. The average for warmth exceeded 3 for the “social” and “kind” items. On the other hand, the score for “emotion” was below 3, suggesting that the participants felt that the android avatar was somewhat emotionless. The average score for competence exceeded 3 for all three items; the mean score exceeded 4 for looking “capable” and “knowledgeable.” For discomfort, the mean was less than 3 for both items. The mean for all four educational effect items exceeded 3.

**TABLE 1:** Questionnaire items for large scale field experiment.

No.	Category	Item	M	SD
1	Anthropomorphism	This robot looks almost human	4.36	0.81
2	Anthropomorphism	This is a robot, but I feel I could mistake it as a human	3.28	1.19
3	Anthropomorphism	This robot moves in a way that is very close to that of a human	3.32	0.97
4	Warmth	This robot seems to have emotions	2.79	1.05
5	Warmth	This robot is social	3.51	1.00
6	Warmth	This robot seems kind	3.16	1.07
7	Competence	This robot looks capable	4.16	0.90
8	Competence	This robot seems knowledgeable	4.13	1.00
9	Competence	This robot seems trustworthy	3.49	1.03
10	Discomfort	This robot is scary	2.98	1.10
11	Discomfort	This robot looks dangerous	2.08	0.99
12	Educational effect	This robot seemed to be looking us	3.37	1.27
13	Educational effect	I was able to concentrate on this lecture	3.87	0.96
14	Educational effect	I could listen to this lecture without losing motivation until the end	3.67	0.99
15	Educational effect	This lecture was easy to understand	4.11	0.82

Based on these results, a covariance structure analysis was conducted to clarify the relationship between anthropomorphism, warmth, competence, discomfort and educational effect. The indicators for anthropomorphism and educational effects were not taken from a single previous study. Therefore, a factor analysis was performed for them, respectively. The maximum likelihood method was used for factor extraction. For anthropomorphism, the analysis revealed one factor (with cumulative contribution rates of 41.189%) with eigenvalues greater than one and factor loadings exceeding 0.35 (Table 2). Chronbach’s alpha was 0.650. For educational effect, the analysis revealed one factor (with cumulative contribution rates of 49.692%) with factor loadings exceeding 0.35 for three items (item13 = 0.897, item14 = 0.823, item15 = 0.676) and below 0.35 for one item (item12 = 0.217). Therefore, we removed item12 from the educational effect. The analysis for educational effect without item12 revealed one factor (with cumulative contribution rates of 64.699%) with eigenvalues greater than one and factor loadings exceeding 0.35 (Table 3). Chronbach’s alpha was 0.839. The warmth, competence, and discomfort were partially adopted from the same paper, so that only Chronbach’s alphas were calculated (warmth = 0.724, competence = 0.738, discomfort = 0.569). Since all of them exceeded 0.5, the average values of each were used for this analysis.

**TABLE 2:** Factor structure of anthropomorphism.

Variables	1st
item1	0.481
item2	0.807
item3	0.481

**TABLE 3:** Factor structure of educational effect.

Variables	1st
item13	0.900
item14	0.819
item15	0.677

**FIGURE 6:**
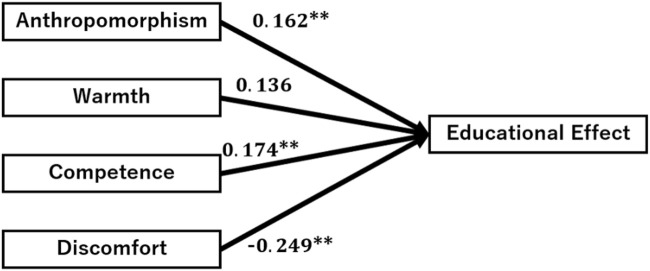
Results of the path analysis for investigating the effect of each variable on the educational effect.

Based on these results, covariance structure analysis with AMOS (a path analysis) was conducted to investigate how the four categories, namely, anthropomorphism, warmth, competence, and discomfort, affect the educational effects. The results of the path analysis for each educational effect are shown in Figure 6. The model fit was GFI=0.872 , CFI=0.632 , AIC=135.111 . The coefficients for educational effect from anthropomorphism. competence, and discomfort were significant (p<0.05) . Considereing the positive and negative value of coeffeicients, the students’ perception of android’s anthropomorphism and competence has a positive impact on the subjective educational effects, and the standardized coefficient were anthropomorphism: 0.162, competence:0.174. The students’ perception of discomfort has a negative impact for educational effects and standardized coefficient was −0.249.

## 6 Discussion

Study 1 compared the educational effects based on the surprise test scores and subjective evaluations of a remote lecture given by a speaker via a videoconferencing system and through an android avatar. The results showed that the use of android avatar scored significantly higher in the three indicators (“I felt this robot (speaker) was looking at us,” “I could concentrate on this lecture,” and “I could listen to this lecture without losing motivation until the end”) used as subjective measures of educational effect. This suggests that the use of an android avatar in distance learning made the audience experience higher subjective educational effects than when a conventional videoconferencing systems was used. On the other hand, the results of the surprise test were slightly higher on an average for the android avatar condition, but this difference was not significant. The full score for the surprise test was 9, whereas the result for the videoconferencing system condition was M=7.89 , SD=1.03 and that for the android avatar condition was M=8.29 , SD=1.00 , both of which were high. Summing up both conditions, M=8.11 , SD=1.03 . Thus, it is suspected that a ceiling effect may have occurred (M+SD=9.14>9) , and this surprise test may have been too easy for the participants. Thus, these results demonstrate the effectiveness of using android avatars to hold lectures.

In Study 2, a large-scale field experiment was conducted to examine the factors that increase the educational effects of android avatars. A lecture was conducted by an android avatar for high school students and the relationship between the educational effects and the four latent variables, namely, anthropomorphism, warmth, competence, and discomfort, on the perception of the android avatar were investigated from the answers to the questionnaire and performing a path analysis. The results of the questionnaire showed that all three items for anthropomorphism, two of the three items for warmth, and all three items for competence were rated above the median value of 3. The discomfort scores were below 3 for both items, suggesting that the audience did not feel particularly uncomfortable with the android. Furthermore, the results exceeded 3 for all four items for the educational effects, indicating that the participants’ subjective experience of the educational effects were high, again confirming the results of Study 1 as an absolute evaluation. The Path analysis was conducted to clarify the relationship between anthropomorphism, warmth, competence, discomfort, and educational effect. Anthropomorphism, competence, and discomfort were significant paths for educational effect. This means that the students’ perception of android’s anthropomorphism and competence has a positive impact, and discomfort has a negative impact on subjective educational effects.

Additionally, this study has the advantage of conducting two experiments, one to determine the effect of the avatar’s embodiment on the educational effects, and the other to determine what improves the educational effects in distance learning by android avatar. The fact that the discussion is based on the laboratory experiment and the large-scale field experiment adds to the value of this study and the persuasiveness of the results. Thus, this study has established the potential effectiveness of android avatars in educational settings, especially when social presence is an essential component of a better educational experience that is difficult to achieve in the distance learning environment.

The difficulty in researching robot-assisted education lies in the difficulty of simple comparisons due to a variety of factors, such as appearance and voice. In fact, Velentza et al. (2021) compared with humans, but it was insufficient in terms of such control. Therefore, in this study, we conducted an experiment using a Geminoid with the same appearance as the individual. Hoshikawa et al. (2015) also conducted experiments using Geminoid and controlled such factors as much as possible. The result of study 1 in this study supports Hoshikawa et al.‘s study that the android is better than video in lectures. On the other hand, although the study by Hoshikawa et al. shows the importance of human-like functions, it does not clarify the relationship between other evaluations of android and educational effects. Hashimoto et al. (2011) did not fully investigate factors that influence the educational effect of android, and Abildgaard and Scharfe (2012) also focused on factors other than android, such as seating position and gender. The result of study 2 of this study, which investigated and analyzed in detail the relationship between the evaluation of the android and the educational effect, which had not been done in such previous studies, revealed the factors that influence the educational effect of anthropomorphism, competence, and discomfort.

One limitation of this study is that the high school students in Study 2 had studied about Prof. Hiroshi Ishiguro and his works before the lecture. It is possible that this prior learning may have reduced the surprise and resistance to the android. Another point to note is the content of the lecture. The title of the lecture in this study was “Avatars and Future Society” and it included content related to avatars. Thus, the possibility that the content itself affected the concentration and motivation for the lecture given by the android avatar rather than a lecture with content unrelated to avatars or science cannot be ruled out. In addition, because the android’s appearance was modeled after a university professor, science-related content may have influenced the credibility and persuasiveness of the lecture itself as perceived by the audience. Study 2 also showed higher results for the subjective evaluation of competence and knowledge compared to the other items. Previous study has also discussed the differences in results depending on the educational content (Hashimoto et al., 2011). Therefore, we aim to confirm the results of this study using different educational contents with an android avatar who is not a renowned professor in our future studies. The long-term effects of android avatar lectures have not yet been verified as well, and further experiment is needed. Nevertheless, this study is important in that it clarifies whether an android avatar is effective in a single lecture and what influences these effects.

## 7 Conclusion

Two studies were conducted to investigate the educational effects of using an android avatar in distance learning. In Study 1, a laboratory experiment was performed, comparing a lecture given using a videoconferencing system with that given by an android avatar (actually recorded) and it was confirmed that significant high feeling of being seen, focused and motivated by the lecture given by the android avatar than videoconferencingsystem. In Study 2, a large-scale field experiment with high school students was conducted to investigate the factors that enhance the educational effects of the android avatar. The results showed that students’ perception of android’s anthropomorphism and competence has a positive impact, and discomfort has a negative impact on the educational effects of the android lecture. In this study, a Geminoid having the same appearance as that of a well-known professor was used. Thus, further investigation is needed to generalize the results. On the other hand, most androids with the same appearance as a specific person have been created for well-known people, and we believe that the results of this study are valid in such cases.

## Data Availability

The datasets presented in this article are not readily available because it will be made shared within the consent of the research participant. Requests to access the datasets should be directed to kodani.naoki@irl.sys.es.osaka-u.ac.jp.
